# Acupoints for Tension-Type Headache: A Literature Study Based on Data Mining Technology

**DOI:** 10.1155/2021/5567697

**Published:** 2021-03-12

**Authors:** Lingyun Lu, Qian Wen, Xinyu Hao, Qianhua Zheng, Ying Li, Ning Li

**Affiliations:** ^1^Department of Integrated Traditional Chinese and Western Medicine, West China Hospital, Sichuan University, Chengdu, Sichuan Province, China; ^2^College of Acupuncture-Moxibustion and Tuina, Chengdu University of Traditional Chinese Medicine, Chengdu, Sichuan Province, China; ^3^Graduate School, Chengdu University of Traditional Chinese Medicine, Chengdu, Sichuan Province, China

## Abstract

**Objectives:**

This study aimed to explore the characteristics and principles of acupoints, which were applied for treating tension-type headache (TTH).

**Methods:**

Four databases were searched for the literature studies of treating TTH with acupuncture and moxibustion up to September 1, 2020. Titles, journals, authors, key words, interventions, main acupoints, and outcomes of the included literature studies were extracted and inputted into the self-established Data Excavation Platform of Acupoint Specificity for analysis.

**Results:**

In total, 128 papers containing 137 prescriptions, 89 meridian acupoints, and 7 extraordinary acupoints of treating TTH with acupuncture and moxibustion were included. The total frequency of acupoints' application was 763 times. Fengchi (GB20), Baihui (GV20), Taiyang (EX-HN5), Hegu (LI4), and Taichong (LR3) were used most frequently. The acupoints in Yang meridians were utilized more than those in Yin meridians (66.1% vs. 17.8%), and the acupoints in the Gallbladder Meridian of Foot Shaoyang were applied most commonly. 59.9% (457/763) of the applied acupoints were on the head, face, and neck, and 31.7% (242/763) were on the four limbs. Additionally, the proportion of specific acupoints' application was 78.2% (597/763).

**Conclusions:**

The prescription of Fengchi (GB20), Baihui (GV20), Taiyang (EX-HN5), Hegu (LI4), and Taichong (LR3) might be relatively reasonable in clinical practices of treating TTH with acupuncture, which should be verified in further studies.

## 1. Introduction

Headache disorders become one of the most common diseases on the nervous system worldwide. The fact sheet of the World Health Organization (WHO) in April 2016 reported that half to three-quarters of adults aged 18–65 years have suffered from headache within one year. Tension-type headache (TTH), the most prevalent type of headache disorders, is characterized by a typically bilateral pain of pressing or tightening in quality, mild to moderate in intensity, and not becoming worse with routine physical activity [1]. According to the Global Burden of Disease study, TTH has become the second most common chronic disease worldwide, affecting more than 10% of the world population [2]. The unclear pathogenesis makes TTH difficult to be cured. Despite simple analgesics and NSAIDs which are the mainstays in the acute therapy of TTH, there are some unpleasant gastrointestinal side effects associated with these acute drugs, including ulcers, hemorrhage, and perforation of the gastrointestinal tract [3, 4]. Moreover, frequent and excessive use of analgesics in acute attacks may lead to a risk of medication-overuse headache [5].

According to the guideline developed by the European Federation of Neurological Societies (EFNS), nondrug management should be considered in TTH treatment [6]. As a traditional treatment technique inherited thousands of years, acupuncture has been used for various diseases around the world. Previous meta-analyses and systematic reviews revealed that acupuncture was an effective therapy in treating pain disorders, such as tension-type headache [1], lateral elbow pain [7], labour pain [8], low back pain [9], migraine prophylaxis [10], shoulder pain [11], and peripheral joint osteoarthritis [12]. The systematic reviews from the Cochrane Library conclude that acupuncture is a valuable nonpharmacological tool in patients with frequent episodic or chronic TTH [1, 13], and it appears to be effective and safe for TTH [14, 15].

In traditional Chinese medicine (TCM) theories, the proper selection of acupoints contributes to a significant therapeutic effect. However, the selection methods of acupoints for the same disease are various from different ideas and experiences of the clinical acupuncturists. Nowadays, using modern data mining techniques to find out the principle of acupoint selections and ascertain the optimal combination of acupoints to specific diseases is not only necessary in acupuncture clinical practice but also beneficial to the development of acupuncture from traditional empirical medicine to evidence-based medicine (EBM).

This literature study aimed to explore the characteristics and association rules of acupoints used for treating TTH and to provide a relatively standard prescription for the acupuncture treatment.

## 2. Methods

### 2.1. Search Strategy

Three Chinese databases and an English database (i.e., China National Knowledge Infrastructure, Chinese Biomedicine Database, Wanfang Data, and PubMed) were searched for the literature studies of treating TTH with acupuncture and moxibustion up to September 1, 2020. Language was restricted to English and Chinese. The search terms were as follows: tension-type headache, acupuncture, moxibustion, and their variations. The search strategy is included in Table 1.

### 2.2. Selection Criteria

#### 2.2.1. Types of Studies

Clinical trials aimed to evaluate the effectiveness of treating TTH with acupuncture or moxibustion, with or without randomization, and/or control could be included, whereas the following types such as animal experiments, case reports, reviews, meta-analyses, and systematic reviews should be excluded.

#### 2.2.2. Participants

Participants in the included studies must be diagnosed with TTH, and the minimum sample size should be ten in each group. However, the studies enrolling the participants who were not diagnosed as TTH according to the International Classification of Headache Disorders should be ruled out.

#### 2.2.3. Intervention

Acupuncture and/or moxibustion must be involved in the treatments of TTH with or without additional interventions (e.g., Chinese herb, Western medicine, or other physical therapies) in the included studies. The studies without using needle insertion (e.g., laser stimulating) should be excluded.

#### 2.2.4. Outcomes

At least one clinical outcome measurement related to TTH must be reported in the included studies, such as duration, frequency, and pain intensity of headache. The controlled trials could be included if the patients treated with acupuncture gained more benefits than those who did not get acupuncture therapy. The studies should be excluded if data were published duplicately or only laboratory parameters were reported.

### 2.3. Screening Process

Firstly, potential literature studies were preidentified by LL to exclude those which were obviously irrelevant (e.g., animal experiments, case reports, reviews, meta-analyses, and systematic reviews). Secondly, full texts of remained studies were obtained and screened again by LL and QW. Thirdly, eligible studies were double-checked based on the above selection criteria by XH and QZ separately. Disagreements were solved by discussion.

### 2.4. Data Extraction

Information of the study, such as titles, journals, authors, key words, interventions, main acupoints, and outcomes, was extracted and inputted into the self-established Data Excavation Platform of Acupoint Specificity (Copyright Registration number: 2009SR014647) [16]. If there were several prescriptions in the study, all of the prescriptions of main acupoints should be extracted in different items. The premise is that the therapeutic effect of the acupuncture treatments must be better compared with nonacupuncture treatments.

### 2.5. Data Processing

In the Data Excavation Platform of Acupoint Specificity (Copyright Registration number: 2009SR014647), data related to selection and combination of acupoints could be calculated and analyzed according to the multilevel association rules and the frequent pattern growth (FP-growth) algorithm [17]. FP-growth is one of the most classic and efficient algorithm of frequent itemset mining. In our platform, it was proceeded in two steps: (1) establishing a FP-tree, which was an extended prefix-tree structure for storing acupoint prescriptions about frequent patterns (i.e., acupoint combinations); (2) mining the complete set of acupoint combinations by pattern fragment growth [17].

In the analysis of acupoint combinations, support and confidence were applied. Mathematically, support was the fraction of the total number of transactions in which the itemset occurred, measuring the statistical significance of association rules in the whole dataset. Confidence was the conditional probability of the occurrence of consequent, given the antecedent, reflecting the credibility degree of association rules [18]. In brief, the support of *A* ⟶ *B* indicated that the prescriptions containing both acupoint *A* and acupoint *B* account for the total ones; and the confidence of *A* ⟶ *B* displayed that the prescriptions containing both acupoint *A* and acupoint *B* account for the ones containing acupoint *A*.

## 3. Results

### 3.1. Search Results and Profile of Prescriptions

**Figure 1:**
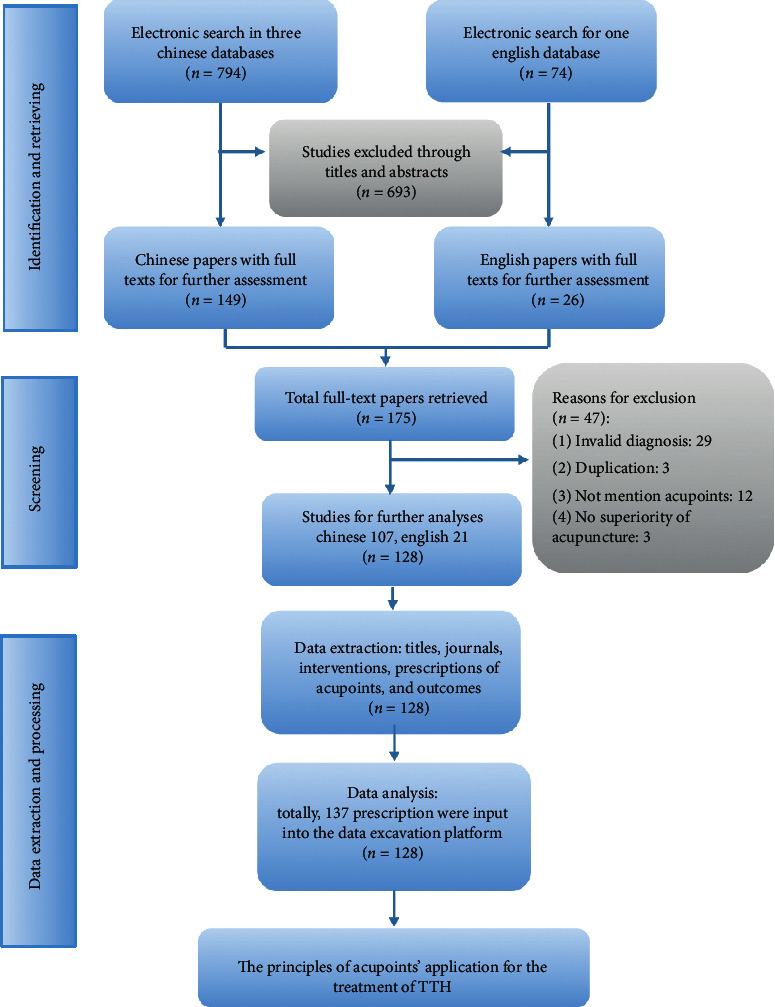
Process of filtering the literature studies and extracting the prescriptions.

In total, 868 records were identified. After screening and evaluation, 137 prescriptions of the main acupoints from 128 records were included in this study. The process of filtering the literature studies and extracting the prescriptions is outlined in Figure 1.

### 3.2. Application of Acupoints

In 137 prescriptions, 89 meridian acupoints and 7 extraordinary acupoints were recorded for 763 times in TTH acupuncture and/or moxibustion treatments. Twenty acupoints applied most frequently are listed in Table 2 in the descending order, and Fengchi (GB20), Baihui (GV20), Taiyang (EX-HN5), Hegu (LI4), and Taichong (LR3) are the top 5 used acupoints.

### 3.3. Association of Meridians and Acupoints

Acupoints could be divided into two categories: meridian acupoints and extraordinary acupoints. In this part, we only discuss the meridian acupoints belonging to the fourteen meridians (the twelve regular meridians, Conception Vessel, and Governor Vessel). Eighty-nine acupoints were distributed in the fourteen meridians. The information of meridian applications is presented in Table 3, including the frequencies and proportions of meridians and the numbers and proportions of the acupoints used in each meridian. From the results of meridians' application, the acupoints in the Gall Bladder Meridian of Foot Shaoyang were applied most frequently. According to the Yin and Yang classification of TCM, 66.1% (504/763) Yang meridians and 17.8% (136/763) Yin meridians were utilized in these studies, respectively.

### 3.4. Correlation between Acupoints and Body Parts

The analysis of acupoints' application on different body parts displays the frequencies and proportions of body distributions, the numbers and percentages of the acupoints used on each body part, and frequency of each used acupoint. Acupoints on the head, face, and neck were used most frequently, with 35 acupoints applied 457 times totally. Detailed information is listed in Table 4.

### 3.5. Application of Specific Acupoints

The utilization rate of specific acupoints needs calculating indirectly through the frequency of nonspecific acupoints, which can eliminate the duplication caused by the direct computation of specific acupoint frequency, because some specific acupoints belong to two or more categories. For example, LR3 belongs to both Five-Shu point and Yuan-primary point, and the record of this point will add once in the frequencies of Five-Shu point and Yuan-primary point, respectively. The computational formula is as follows: utilization rate of specific acupoints = ((total frequency − nonspecific acupoints' application frequency)/total frequency) × 100%. We found that the utilization rate of specific acupoints was as high as 78.2% (597/763) and the nonspecific ones was 21.8% (166/763). Convergent acupoints were most widely used among the nine categories of specific ones. The frequencies of different specific acupoint categories are exhibited in Table 5.

### 3.6. Acupoint Combinations

In this part, the support and confidence were applied to measure the effect of acupoint combinations in those prescriptions, which contained two or more acupoints. The results of acupoint combinations are listed in Table 6. The pairwise combinations of Fengchi (GB20), Baihui (GV20), and Taiyang (EX-HN5) had the top three supports, among which GB20 combined with GV20 (support: 40.9%) was the core for TTH treatment. In addition, LI4 combined with GB20 (support: 28.5%; confidence: 79.6%) and LR3 combined with GB20 (support: 23.4%; confidence: 68.1%) reflected the important role of the distant-local combination principle in TTH treatment.

## 4. Discussion

Nowadays, the acupoint prescriptions for TTH are manifold and lack the uniform standard. A total of four main categories of acupoints' selection can be summarized from the previous studies: (1) the unified acupoint prescription (the unified acupoints applied in all acupuncture treatment sessions) [19]; (2) the semiunified acupoints' prescription (a combination of unified main points and additional points chosen via syndromes or symptoms) [20–23]; (3) the quantitative prescription (choosing a certain number of acupoints from a unified acupoints' pool) [24]; and (4) the nonunified prescription (selecting acupoints completely based on syndrome differentiation without unified main acupoints and developing an individual prescription) [25]. Formulating a standard and effective prescription for TTH seems not only beneficial for diminishing confounding factors in explanatory studies from the perspective of EBM but also extremely essential for optimizing treatment regimens and achieving good effects for the acupuncture clinical practice.

Fortunately, the emergence of data mining techniques makes it feasible to extract effective hidden information and correlation of acupoints from the massive data. In recent years, the acupoints applied for poststroke disorder [26], migraine [27], functional diarrhea [28], vertigo [29], perimenopausal syndrome [30], and primary dysmenorrhea [31] have been explored based on data mining technology, and most of the results have been translated into applications of clinical practice and trials. Our study utilized data mining for secondary analysis of literature studies to explore the characteristics of acupoints for TTH treatment, which may contribute to the development of the optimal standard acupoint prescription.

Our results suggested that acupoints in the Yang meridian and distributed on the head, face, and neck might play some important roles in the TTH treatment. Acupoints of the Gall Bladder Meridian of Foot Shaoyang (GB) and Governor Vessel (GV) were applied with high proportions, a majority of which were on the head. In the TCM theory, head is the confluence of all the Yang meridians, and the hyperactivity of Yang and the stasis of Qi are considered to be the main pathogenesis of headache [32]. Yang and Yin meridians provide layers of energy to protect the integrity of the body. Yang distributes outside, while Yin hides inside. Generally, the acute headache is caused by the abnormal distribution of superficial Qi controlled by Yang meridians initially and disrupts Yin meridians as symptoms become chronic [33]. Therefore, stimulating acupoints of Yang meridians can restore the harmony of Yang and Qi to prevent disturbing Yin. Acupoints on the head, face, and neck were applied more frequently than other parts of the body, which reflects the principle of local and nearby acupoints' selection in line with the traditional saying that where there is an acupoint, there is a corresponding indication. In the modern medicine, although the mechanisms of TTH still remain unclear, muscle nociceptors, myofascial tenderness, and muscle contraction have been demonstrated to play roles in the pathophysiology of TTH [34]. Increased pericranial myofascial tissue tenderness and pressure pain hypersensitivity with the prevalent neck pain are the prominent manifestations in TTH patients [35–38]. The locations of tenderness points, also called myofascial trigger points (MTrPs), are consistent with the distribution range of the GB and GV meridians on the head and neck [37]. Therefore, stimulating these acupoints on the head and neck is a direct and symptomatic treatment for TTH.

Fengchi (GB20) and Baihui (GV20) proved to be the core acupoint combination for TTH in our study, which showed pleasant effects in some previous studies around the world [22, 39]. Pericranial muscle abnormal metabolism, caused by inflammation, decreased skeletal muscle blood flow and muscle atrophy and demonstrated a potential peripheral mechanism of TTH [40–44]. Stimulating GB20 can regulate blood flow velocity positively [45, 46] and inhibit hyperalgesia by increasing the number of mast cells and macrophages and eliminating the serum proinflammatory factors (IL-1*β*, IL-6, TNF-*α*, and COX-2) [47]. Moreover, GB20 can improve the electromyographic activity of sternocleidomastoid and trapezius muscles and increase the tolerance to chronic pain [48]. GV20 shows similar properties of anti-inflammatory and blood flow regulation. Acupuncturing GV20 can relieve cerebral vasospasm [49, 50], increase anti-inflammatory cytokine production, and provide pronounced analgesic relief via the release of mesenchymal stem cells [51]. The above underlying mechanism may be the reason why the combination of GB20 and GV20 can treat TTH collaboratively. According to the local and adjacent therapeutic property of acupoints, EX-HE05 can be taken as the key acupoint for TTH treatment theoretically because it is located in the temporalis muscles, where many MTrPs are distributed [37].

In addition to peripheral mechanisms, central sensitization, mediated by the spinal cord and the trigeminal nerve nucleus caudalis [52], may take an important part in the pathogenesis of TTH, provoked by the continuous peripheral nociception inputs from pericranial myofascial tissues [53]. Hegu (LI4) and Taichong (LR3), known as Siguan, are the classical acupoints for relieving pain and inducing resuscitation. Previous studies have found that LI4 and LR3 exerted a neuroprotective role for the central system, manifested as repairing neurons, alleviating nerve inflammation, and inhibiting cell apoptosis [54–56]. Functional magnetic resonance imaging (fMRI) studies also found that stimulating LI4 and LR3 could transform the pain-activated brain regions to the inhibitory state, exert analgesic effects, and relieve physical pain caused by central sensitization [57, 58]. Moreover, psychological factor is also known as an important inducement for TTH [59, 60]. Acupuncture at Siguan acupoints can relieve depression [61], partly via upregulating hippocampal AMPA receptors [62]. Thus, the therapeutic effects of LI4 and LR3 on TTH may be achieved through both physiological and psychological approaches, indicating the remote therapeutic property of acupoints.

Combining the local or nearby acupoints on the head (i.g., GB20, GV20, and EX-HN05) with the distal acupoint on the limbs (i.g., LI04 and LR03) reflects principles of distant-local and upper-lower acupoints' combinations in the acupuncture theory, which has been adopted in several previous trials of good quality [20–22, 39]. Analysis of treatment details applied in a multicentre randomised trial of acupuncture for tension-type headache (ARTTTH) from Germany has shown that GB20, LR3, and LI4 were the high-frequency acupoints, treated in 96%, 97%, and 67% of sessions, respectively [63]. Not only that, the combination of the above five acupoints also shows therapeutic advantages in the treatment of other primary headaches, such as migraine [64], and the application proportions of GB20, GV20, EX-HN5, LI4, and LR3 calculated from 11 studies of migraine are 82%, 55%, 55%, 46%, and 64%, respectively [65].

However, our results should be interpreted with caution due to the following limitations. Firstly, the outcome measurements and acupuncture stimulation parameters of the included studies were not unified, which might make it confused to evaluate the therapeutic effects contributed by acupoints. Secondly, owing to the lack of the studies accorded with EBM methodologies, some self-controlled studies were included in the analyses, which were difficult to evaluate the qualities and might affect the objectivity of the results.

## 5. Conclusions

Our findings provided a reasonable reference of acupoints' selection and combination for TTH. Fengchi (GB20), Baihui (GV20), Taiyang (EX-HN05), Hegu (LI04), and Taichong (LR03) were recommended as the main acupoints for TTH, and the prescription might be conducive to the standardization of treating TTH with acupuncture, which needed to be further verified through clinical trials and mechanism research.

## Figures and Tables

**Table 1:** Search strategy of the literature study.

A. Search strategy to locate “tension-type headache”	#1. tension-type headache [MeSH]
	#2. TTH [tw]
	#3. primary headache [MeSH]
	#4. or/#1-#3

B. Search strategy to locate acupuncture interventions	#5. acupuncture [MeSH]
	#6. acupuncture therapy [MeSH]
	#7. acupuncture points [MeSH]
	#8. body acupuncture [tw]
	#9. electroacupuncture [MeSH]
	#10. electro-acupuncture [tw]
	#11. electrical acupuncture [tw]
	#12. scalp acupuncture
	#13. dry needling
	#14. triggers point [tw]
	#15. moxibustion [MeSH]
	#16. acupoint [tw]
	#17. or/#5-#16

C. Search strategy to locate literature studies for this study	#4 and #17

**Table 2:** The top twenty acupoints for TTH treatment.

Number	Acupoint	Frequency	^*∗*^Proportion (%)
1	Fengchi (GB20)	100	73.0
2	Baihui (GV20)	74	54.0
3	Taiyang (EX-HN5)	68	49.6
4	Hegu (LI04)	49	35.8
5	Taichong (LR03)	47	34.3
6	Touwei (ST08)	35	25.6
7	Shuaigu (GB08)	29	21.2
8	Sanyinjiao (SP06)	22	16.1
9	Zusanli (ST36)	19	13.9
10	Sishencong (EX-HN1)	18	13.1
11	Shenting (GV24)	16	11.7
12	Yintang (EX-HN3)	15	11.0
13	Tianzhu (BL10)	15	11.0
14	Shenmen (HT07)	13	9.5
15	Lieque (LU07)	11	8.0
16	Fengfu (GV16)	11	8.0
17	Neiguan (PC06)	10	7.3
18	Kunlun (BL60)	9	6.6
19	Waiguan (TE05)	9	6.6
20	Shangxing (GV23)	8	5.8

^*∗*^Proportion refers to the percentage that an acupoint frequency accounts for the total frequency of all acupoints.

**Table 3:** Association analysis of meridians and acupoints used in TTH treatment.

Number	Meridian	Frequency	^*∗*^Proportion (%)	Acupoints in each meridian		
				Number	^#^Proportion (%)	Acupoints and their frequency
1	GB	175	23.6	17	20.5	Fengchi (GB20) 100, Shuaigu (GB08) 29, Jianjing (GB21) 7, Yangbai (GB14) 6, Yanglingquan (GB34) 5, Xuanlu (GB05) 4, Wangu (GB12) 4, Zhengying (GB17) 4, Zulinqi (GB41) 4, Hanyan (GB04) 2, Toulinqi (GB15) 2, Naokong (GB19) 2, Xiaxi (GB43) 2, Benshen (GB13) 1, Huantiao (GB30) 1, Xuanzhong (GB39) 1, and Zuqiaoyin (GB44) 1
2	GV	126	17.0	10	12.1	Baihui (GV20) 74, Shenting (GV24) 16, Fengfu (GV16), Shangxing (GV23) 8, Dazhui (GV14) 5, Houding (GV19) 4, Qianding (GV21) 3, Yamen (GV15) 2, Naohu (GV17) 2, and Shuigou (GV26)
3	ST	64	8.6	7	8.4	Touwei (ST08) 35, Zusanli (ST36) 19, Neiting (ST44) 4, Huaroumen (ST24) 2, Fenglong (ST40) 2, Tianshu (ST25) 1, and Wailing (ST26) 1
4	BL	54	7.3	15	18.1	Tianzhu (BL10) 15, Kunlun (BL60) 9, Cuanzhu (BL02) 7, Ganshu (BL18) 4, Tongtian (BL07) 3, Shenmai (BL62) 3, Quchai (BL04) 2, Wuchu (BL05) 2, Chengguang (BL06) 2, Luoque (BL08) 2, Yuzhen (BL09) 1, Fengmen (BL12) 1, Feishu (BL13) 1, Xinshu (BL15) 1, and Pishu (BL20) 1
5	LR	52	7.0	2	2.4	Taichong (LR03) 47 and Xingjian (LR02) 5
6	LI	51	6.9	3	3.6	Hegu (LI04) 49, Shousanli (LI10) 1, and Quchi (LI11) 1
7	SP	25	3.4	2	2.4	Sanyinjiao (SP06) 22 and Xuehai (SP10) 3
8	TE	25	3.4	8	9.6	Waiguan (TE05) 9, Yifeng (TE17) 4, Jiaosun (TE20) 3, Sizhukong (TE23) 3, Zhigou (TE06) 2, Luxi (TE19) 2, Zhongzhu (TE03) 1, and Tianliao (TE15) 1
9	CV	14	1.9	6	7.2	Zhongwan (CV12) 4, Guanyuan (CV04) 3, Qihai (CV06) 3, Juque (CV14) 2, Shuifen (CV09) 1, and Danzhong (CV17) 1
10	HT	13	1.8	1	1.2	Shenmen (HT07) 13
11	PC	12	1.6	2	2.4	Neiguan (PC06) 10 and Daling (PC07) 2
12	LU	11	1.5	1	1.2	Lieque (LU07) 11
13	SI	9	1.2	3	3.6	Houxi (SI03) 7, Jianwaishu (SI14) 1, and Jianzhongshu (SI15) 1
14	KI	9	1.2	3	3.6	Taixi (KI03) 7, Yongquan (KI01) 1, and Yindu (KI19) 1

GB refers to the Gallbladder Meridian of Foot Shaoyang. GV refers to the Governor Vessel. ST stands for the Stomach Meridian of Foot Yangming. BL refers to the Bladder Meridian of Foot Taiyang. LR refers to the Liver Meridian of Foot Jueyin. LI refers to the Large Intestine Meridian of Hand Yangming. SP refers to the Spleen Meridian of Foot Taiyin. TE refers to Triple Energizer of Hand Shaoyang. CV refers to the Conception Vessel. HT refers to the Heart Meridian of Hand Shaoyin. PC refers to the Pericardium Meridian of Hand Jueyin. LU refers to the Lung Meridian of Hand Taiyin. SI refers to the Small Intestine Meridian of Hand Taiyang. KI refers to the Kidney Meridian of Foot Shaoyin. ^*∗*^Proportion means the percentage that a specific meridian frequency accounts for the total frequency of all meridians. ^#^Proportion refers to the percentage that the number of acupoints in a meridian accounts for the total number of meridian acupoints.

**Table 4:** Association analysis of body parts and acupoints used in TTH treatment.

Number	Body part	Frequency	Proportion (%)	Acupoints in each body part		
				Number	Proportion (%)	Selected acupoints and their frequency
1	Head, face, and neck	457	59.9	35	36.5	Fengchi (GB20) 100, Baihui (GV20) 74, Taiyang (EX-HN5) 68, Touwei (ST08) 35, and Shuaigu (GB08) 29
2	Lower limbs	136	17.8	17	17.7	Taichong (LR03) 47, Sanyinjiao (SP06) 22, Zusanli (ST36) 19, Kunlun (BL60) 9, and Taixi (KI03) 7
3	Upper limbs	106	13.9	11	11.5	Hegu (LI04) 49, Shenmen (HT07) 13, Lieque (LU07) 11, Neiguan (PC06) 10, and Waiguan (TE05) 9
4	Back and lumbar	33	4.3	18	18.8	Jianjing (GB21) 7, Dazhui (GV14) 5, Ganshu (BL18) 4, Jianwaishu (SI14) 2, and Jianzhongshu (SI15) 2
5	Chest and abdomen	31	4.1	15	15.6	Zhongwan (CV12) 4, Guanyuan (CV04) 3, Qihai (CV06) 3, Juque (CV14) 2, and Huaroumen (ST24) 2

**Table 5:** Different types of specific acupoints applied in TTH treatment.

Number	Specific acupoint	Frequency	Amount of acupoints	Selected acupoints and their frequency
1	Crossing point	353	30	Fengchi (GB20) 100, Baihui (GV20) 74, Touwei (ST08) 35, Shuaigu (GB08) 29, and Sanyinjiao (SP06) 22
2	Five-Shu point	130	17	Taichong (LR03) 47, Zusanli (ST36) 19, Shenmen (HT07) 13, Kunlun (BL60) 9, Houxi (KI03) 7, and Taixi (KI03) 7
3	Yuan-primary point	118	5	Hegu (LI04) 49, Taichong (LR03) 47, Shenmen (HT07) 13, Taixi (KI03), and Daling (PC07) 2
4	Eight confluent points	44	6	Lieque (LU07) 11, Neiguan (PC06) 10, Waiguan (TE05) 9, Houxi (SI03) 7, and Zulingqi (GB41) 4
5	Luo-connecting point	32	4	Lieque (LU07) 11, Neiguan (PC06) 10, Waiguan (TE05) 9, and Fenglong (ST40) 2
6	Lower He-sea point	24	2	Zusanli (ST36) 19 and Yanglingquan (GB34) 5
7	Eight converging points	11	4	Yanglingquan (GB34) 5, Zhongwan (CV12) 4, Tanzhong (CV17) 1, and Xuanzhong (GB39) 1
8	Front-Mu point	11	5	Zhongwan (CV12) 4, Guanyuan (CV04) 3, Juque (CV14) 2, Tanzhong (CV17) 1, and Tianshu (ST25) 1
9	Back-Shu point	7	4	Ganshu (BL18) 4, Feishu (BL13) 1, Xinshu (BL15) 1, and Pishu (BL20) 1

**Table 6:** The top ten acupoint combinations in TTH treatment.

Number	Combination of acupoints	Support (%)	Confidence (%)
1	Baihui (GV20) ⟶ Fengchi (GB20)	40.9	75.7
2	Fengchi (GB20) ⟶ Taiyang (EX-HN5)	40.1	80.9
3	Taiyang (EX-HN5) ⟶ Baihui (GV20)	32.8	66.2
4	Hegu (LI04) ⟶ Fengchi (GB20)	28.5	79.6
5	Fengchi (GB20) ⟶ Baihui (GV20), Taiyang (EX-HN5)	27.0	82.2
6	Taichong (LR03) ⟶ Fengchi (GB20)	23.4	68.1
7	Taichong (LR03) ⟶ Baihui (GV20)	23.4	68.1
8	Touwei (ST08) ⟶ Fengchi (GB20)	21.9	85.7
9	Taichong (LR03) ⟶ Taiyang (EX-HN5)	21.9	63.8
10	Shuaigu (GB08) ⟶ Fengchi (GB20)	19.7	93.1

Support of *A* ⟶ *B* indicated that the prescriptions containing both acupoint *A* and acupoint *B* account for the total ones; confidence of *A* ⟶ *B* displayed that the prescriptions containing both acupoint *A* and acupoint *B* account for the ones containing acupoint *A*.
